# 1-Methyl-5-(4-methyl­phen­yl)-3-oxo­cyclo­hexane-1-carbonitrile

**DOI:** 10.1107/S1600536808009070

**Published:** 2008-04-10

**Authors:** R. T. Sabapathy Mohan, S. Kamatchi, M. Subramanyam, A. Thiruvalluvar, A. Linden

**Affiliations:** a Department of Chemistry, Annamalai University, Annamalai Nagar 608 002, Tamil Nadu, India; b PG Research Department of Physics, Rajah Serfoji Government College (Autonomous), Thanjavur 613 005, Tamil Nadu, India; c Institute of Organic Chemistry, University of Zürich, Winterthurerstrasse 190, CH-8057 Zürich, Switzerland

## Abstract

In the title mol­ecule, C_15_H_17_NO, the cyclo­hexane ring adopts a chair conformation. The cyano and methyl groups at position 1 have axial and equatorial orientations, respectively. The benzene ring has an equatorial orientation. A C—H⋯π inter­action involving the benzene ring is found in the crystal structure.

## Related literature

Subramanyam *et al.* (2007 ▶) have reported the crystal structure of 3-cyano-3-methyl-5-phenyl­cyclo­hexane, in which the cyclo­hexane ring adopts a chair conformation.
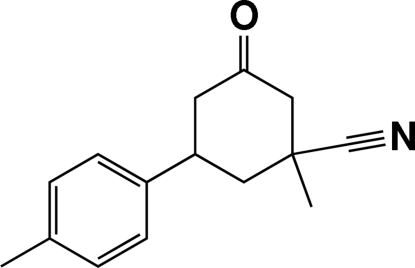


## Experimental

### 

#### Crystal data

C_15_H_17_NO
*M* *_r_* = 227.30
Monoclinic, 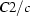

*a* = 23.4475 (5) Å
*b* = 6.0370 (1) Å
*c* = 21.0740 (5) Å
β = 123.267 (1)°
*V* = 2494.22 (9) Å^3^
*Z* = 8
Mo *K*α radiation
μ = 0.07 mm^−1^
*T* = 160 (1) K
0.25 × 0.20 × 0.10 mm

#### Data collection

Nonius KappaCCD area-detector diffractometer
Absorption correction: none
37687 measured reflections
3640 independent reflections
2682 reflections with *I* > 2σ(*I*)
*R*_int_ = 0.054

#### Refinement

*R*[*F* ^2^ > 2σ(*F* ^2^)] = 0.052
*wR*(*F* ^2^) = 0.170
*S* = 1.08
3640 reflections
154 parameters
H-atom parameters constrained
Δρ_max_ = 0.42 e Å^−3^
Δρ_min_ = −0.27 e Å^−3^

### 

Data collection: *COLLECT* (Nonius, 2000 ▶); cell refinement: *DENZO-SMN* (Otwinowski & Minor, 1997 ▶); data reduction: *DENZO-SMN* and *SCALEPACK* (Otwinowski & Minor, 1997 ▶); program(s) used to solve structure: *SHELXS97* (Sheldrick, 2008 ▶); program(s) used to refine structure: *SHELXL97* (Sheldrick, 2008 ▶); molecular graphics: *ORTEP-3* (Farrugia, 1997 ▶); software used to prepare material for publication: *PLATON* (Spek, 2003 ▶).

## Supplementary Material

Crystal structure: contains datablocks global, I. DOI: 10.1107/S1600536808009070/wn2249sup1.cif

Structure factors: contains datablocks I. DOI: 10.1107/S1600536808009070/wn2249Isup2.hkl

Additional supplementary materials:  crystallographic information; 3D view; checkCIF report

## Figures and Tables

**Table 1:** Hydrogen-bond geometry (Å, °)

*D*—H⋯*A*	*D*—H	H⋯*A*	*D*⋯*A*	*D*—H⋯*A*
C4—H4*A*⋯*Cg*^i^	0.99	2.61	3.5425 (15)	157

Symmetry code: (i) 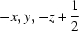. *Cg *is the centroid of the benzene ring.

**Fig. 1.**
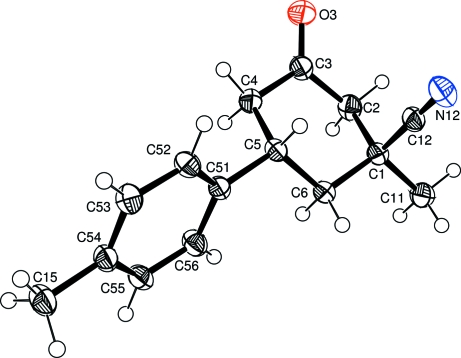
The molecular structure of the title compound, showing the atom-numbering scheme and displacement ellipsoids drawn at the 50% probability level. Hydrogen atoms are represented by spheres of arbitrary radius.

## supplementary crystallographic information

### Comment

The title compound has been analysed as part of our crystallographic studies on substituted cyclohexanes (Subramanyam *et al.*,  2007). Its molecular structure, with atomic numbering scheme, is shown in Fig. 1. The cyclohexane ring adopts a chair conformation. The cyano group and the methyl group at position 1 have axial and equatorial orientations respectively. The benzene ring at position 5 has an equatorial orientation. A C4—H4A···π(-*x*, *y*, 1/2 - *z*) interaction involving the benzene ring is found in the structure. No classical hydrogen bonds are found in the crystal structure.

### Experimental

A mixture of 5-4'-methylphenyl-3-methylcyclohex-2-enone (4.00 g, 0.02 mol), potassium cyanide (2.60 g, 0.04 mol), ammonium chloride (1.59 g, 0.03 mol), dimethylformamide (50 ml) and water (2 ml) was heated with stirring for 16-18 h at 353 K. The reaction mixture was cooled to room temperature and poured into water. The product was extracted with CH_2_Cl_2_ (3x10 ml) and the organic layer was dried, evaporated and purified by column chromatography (hexane-EtOAc, 4.5:1 *v*/*v*). The yield of the isolated product was 3.40 g (75%).

### Refinement

H atoms were positioned geometrically and allowed to ride on their parent atoms, with C—H = 0.95 Å for Csp^2^, 0.98 Å for methyl C, 0.99 Å for methylene C and 1.00 Å for methine C; U_iso_(H) = xU_eq_(carrier atom), where x = 1.5 for methyl and 1.2 for all other C atoms

### Crystal data

**Table:**

C_15_H_17_NO	*F*_000_ = 976
*M**_r_* = 227.30	*D*_x_ = 1.211 Mg m^−^^3^
Monoclinic, *C*2/*c*	Melting point: 376 K
Hall symbol: -C 2yc	Mo *K*α radiation λ = 0.71073 Å
*a* = 23.4475 (5) Å	Cell parameters from 3933 reflections
*b* = 6.0370 (1) Å	θ = 2.0–30.0º
*c* = 21.0740 (5) Å	µ = 0.08 mm^−^^1^
β = 123.267 (1)º	*T* = 160 (1) K
*V* = 2494.22 (9) Å^3^	Tablet, colourless
*Z* = 8	0.25 × 0.20 × 0.10 mm

### Data collection

**Table:**

Nonius KappaCCD area-detector diffractometer	3640 independent reflections
Radiation source: Nonius FR590 sealed tube generator	2682 reflections with *I* > 2σ(*I*)
Monochromator: horizontally mounted graphite crystal	*R*_int_ = 0.054
Detector resolution: 9 pixels mm^-1^	θ_max_ = 30.1º
*T* = 160(1) K	θ_min_ = 2.1º
φ and ω scans with κ offsets	*h* = 0→32
Absorption correction: none	*k* = 0→8
37687 measured reflections	*l* = −29→24

### Refinement

**Table:**

Refinement on *F*^2^	Secondary atom site location: difference Fourier map
Least-squares matrix: full	Hydrogen site location: inferred from neighbouring sites
*R*[*F*^2^ > 2σ(*F*^2^)] = 0.052	H-atom parameters constrained
*wR*(*F*^2^) = 0.170	*w* = 1/[σ^2^(*F*_o_^2^) + (0.0889*P*)^2^ + 1.0674*P*] where *P* = (*F*_o_^2^ + 2*F*_c_^2^)/3
*S* = 1.08	(Δ/σ)_max_ < 0.001
3640 reflections	Δρ_max_ = 0.42 e Å^−^^3^
154 parameters	Δρ_min_ = −0.27 e Å^−^^3^
Primary atom site location: structure-invariant direct methods	Extinction correction: none

### Special details

**Table:**

Experimental. Solvent used: ? Cooling Device: Oxford Cryosystems Cryostream 700 Crystal mount: glued on a glass fibre Mosaicity (°.): 0.608 (1) Frames collected: 469 Seconds exposure per frame: 68 Degrees rotation per frame: 1.7 Crystal-Detector distance (mm): 30.0
Geometry. Bond distances, angles *etc*. have been calculated using the rounded fractional coordinates. All su's are estimated from the variances of the (full) variance-covariance matrix. The cell e.s.d.'s are taken into account in the estimation of distances, angles and torsion angles
Refinement. Refinement of *F*^2^ against ALL reflections. The weighted *R*-factor *wR* and goodness of fit *S* are based on *F*^2^, conventional *R*-factors *R* are based on *F*, with *F* set to zero for negative *F*^2^. The threshold expression of *F*^2^ > σ(*F*^2^) is used only for calculating *R*-factors(gt) *etc*. and is not relevant to the choice of reflections for refinement. *R*-factors based on *F*^2^ are statistically about twice as large as those based on *F*, and *R*- factors based on ALL data will be even larger.

### Fractional atomic coordinates and isotropic or equivalent isotropic displacement parameters (Å^2^)

**Table:**

	*x*	*y*	*z*	*U*_iso_*/*U*_eq_	
O3	−0.15528 (6)	0.46344 (19)	0.25177 (7)	0.0415 (4)	
N12	−0.08326 (7)	0.6151 (2)	0.47679 (8)	0.0396 (4)	
C1	−0.11894 (7)	0.9214 (2)	0.37288 (8)	0.0257 (4)	
C2	−0.16638 (7)	0.8175 (3)	0.29352 (8)	0.0301 (4)	
C3	−0.13059 (7)	0.6453 (2)	0.27576 (7)	0.0284 (4)	
C4	−0.06263 (7)	0.7130 (2)	0.28980 (8)	0.0285 (4)	
C5	−0.01555 (6)	0.8211 (2)	0.36851 (7)	0.0235 (3)	
C6	−0.05364 (7)	1.0065 (2)	0.37989 (8)	0.0257 (4)	
C11	−0.15578 (8)	1.1085 (3)	0.38596 (10)	0.0358 (5)	
C12	−0.09953 (7)	0.7479 (2)	0.43103 (8)	0.0283 (4)	
C15	0.24199 (7)	1.0891 (3)	0.40745 (9)	0.0366 (5)	
C51	0.05077 (6)	0.8959 (2)	0.37806 (7)	0.0234 (3)	
C52	0.10525 (7)	0.7488 (2)	0.40770 (7)	0.0268 (4)	
C53	0.16606 (7)	0.8088 (2)	0.41545 (8)	0.0288 (4)	
C54	0.17508 (7)	1.0191 (3)	0.39554 (8)	0.0283 (4)	
C55	0.12020 (7)	1.1653 (2)	0.36508 (9)	0.0321 (4)	
C56	0.05907 (7)	1.1049 (2)	0.35635 (9)	0.0308 (4)	
H2A	−0.20592	0.74844	0.29089	0.0361*	
H2B	−0.18388	0.93583	0.25469	0.0361*	
H4A	−0.07029	0.81889	0.24998	0.0341*	
H4B	−0.03970	0.58069	0.28600	0.0341*	
H5	−0.00387	0.70502	0.40771	0.0282*	
H6A	−0.06599	1.12360	0.34155	0.0308*	
H6B	−0.02313	1.07305	0.43072	0.0308*	
H11A	−0.16873	1.22489	0.34797	0.0537*	
H11B	−0.12531	1.17052	0.43686	0.0537*	
H11C	−0.19680	1.04942	0.38141	0.0537*	
H15A	0.27435	0.96550	0.42915	0.0549*	
H15B	0.26039	1.21551	0.44228	0.0549*	
H15C	0.23475	1.13160	0.35866	0.0549*	
H52	0.10086	0.60508	0.42290	0.0321*	
H53	0.20209	0.70396	0.43470	0.0345*	
H55	0.12464	1.30917	0.34997	0.0384*	
H56	0.02242	1.20775	0.33526	0.0370*	

### Atomic displacement parameters (Å^2^)

**Table:**

	*U*^11^	*U*^22^	*U*^33^	*U*^12^	*U*^13^	*U*^23^
O3	0.0412 (6)	0.0397 (6)	0.0472 (7)	−0.0142 (5)	0.0266 (5)	−0.0148 (5)
N12	0.0409 (7)	0.0425 (8)	0.0396 (7)	−0.0060 (6)	0.0248 (6)	0.0051 (6)
C1	0.0254 (6)	0.0250 (6)	0.0306 (7)	−0.0027 (5)	0.0178 (6)	−0.0022 (5)
C2	0.0233 (6)	0.0366 (8)	0.0285 (7)	−0.0015 (6)	0.0130 (5)	−0.0014 (6)
C3	0.0273 (7)	0.0332 (8)	0.0231 (7)	−0.0052 (5)	0.0129 (5)	−0.0028 (5)
C4	0.0283 (7)	0.0303 (7)	0.0304 (7)	−0.0032 (5)	0.0184 (6)	−0.0045 (5)
C5	0.0224 (6)	0.0230 (6)	0.0265 (6)	−0.0008 (5)	0.0144 (5)	0.0021 (5)
C6	0.0258 (6)	0.0246 (7)	0.0301 (7)	−0.0042 (5)	0.0176 (6)	−0.0030 (5)
C11	0.0364 (8)	0.0305 (8)	0.0515 (9)	−0.0015 (6)	0.0311 (7)	−0.0045 (7)
C12	0.0258 (6)	0.0329 (7)	0.0303 (7)	−0.0071 (5)	0.0181 (6)	−0.0048 (6)
C15	0.0275 (7)	0.0443 (9)	0.0427 (9)	−0.0070 (6)	0.0222 (7)	−0.0033 (7)
C51	0.0229 (6)	0.0248 (6)	0.0241 (6)	−0.0018 (5)	0.0140 (5)	0.0003 (5)
C52	0.0258 (6)	0.0264 (7)	0.0272 (7)	−0.0003 (5)	0.0140 (5)	0.0040 (5)
C53	0.0232 (6)	0.0332 (7)	0.0281 (7)	0.0030 (5)	0.0128 (5)	0.0034 (6)
C54	0.0249 (6)	0.0340 (7)	0.0289 (7)	−0.0052 (5)	0.0167 (6)	−0.0035 (5)
C55	0.0346 (7)	0.0252 (7)	0.0450 (9)	−0.0036 (6)	0.0273 (7)	0.0009 (6)
C56	0.0296 (7)	0.0258 (7)	0.0428 (8)	0.0033 (5)	0.0235 (7)	0.0056 (6)

### Geometric parameters (Å, °)

**Table:**

O3—C3	1.2145 (17)	C2—H2A	0.9900
N12—C12	1.1466 (19)	C2—H2B	0.9900
C1—C2	1.545 (2)	C4—H4A	0.9900
C1—C6	1.543 (3)	C4—H4B	0.9900
C1—C11	1.536 (3)	C5—H5	1.0000
C1—C12	1.4812 (19)	C6—H6A	0.9900
C2—C3	1.507 (2)	C6—H6B	0.9900
C3—C4	1.509 (3)	C11—H11A	0.9800
C4—C5	1.5450 (19)	C11—H11B	0.9800
C5—C6	1.531 (2)	C11—H11C	0.9800
C5—C51	1.523 (2)	C15—H15A	0.9800
C15—C54	1.507 (3)	C15—H15B	0.9800
C51—C52	1.391 (2)	C15—H15C	0.9800
C51—C56	1.3917 (18)	C52—H52	0.9500
C52—C53	1.391 (3)	C53—H53	0.9500
C53—C54	1.389 (2)	C55—H55	0.9500
C54—C55	1.393 (2)	C56—H56	0.9500
C55—C56	1.390 (3)		
			
O3···H6A^i^	2.8000	H4A···C55^vii^	2.9200
O3···H11A^i^	2.6400	H4A···C56^vii^	2.9500
O3···H15C^ii^	2.8500	H4B···C52	3.1000
O3···H55^ii^	2.7700	H4B···H56^i^	2.5700
N12···H11B^i^	2.8200	H5···C12	2.5600
N12···H5^iii^	2.8900	H5···H52	2.3700
N12···H6B^iv^	2.8700	H5···N12^iii^	2.8900
N12···H52^iii^	2.7100	H6A···O3^ix^	2.8000
C4···C12	3.532 (2)	H6A···C56	2.7800
C12···C4	3.532 (2)	H6A···H2B	2.5900
C12···C15^v^	3.598 (3)	H6A···H11A	2.5600
C15···C12^vi^	3.598 (3)	H6A···H56	2.2100
C6···H56	2.7200	H6B···C56	3.0900
C12···H5	2.5600	H6B···H11B	2.5400
C12···H6B^iv^	2.9600	H6B···N12^iv^	2.8700
C15···H2B^vii^	3.0500	H6B···C12^iv^	2.9600
C51···H4A^vii^	3.0100	H11A···O3^ix^	2.6400
C52···H4B	3.1000	H11A···H2B	2.5000
C52···H55^i^	3.0600	H11A···H6A	2.5600
C52···H4A^vii^	2.9800	H11B···N12^ix^	2.8200
C52···H11B^iv^	3.0800	H11B···H6B	2.5400
C53···H4A^vii^	2.9300	H11B···C52^iv^	3.0800
C53···H53^viii^	2.9700	H11C···H2A	2.5600
C54···H4A^vii^	2.9400	H15A···H53	2.3700
C55···H52^ix^	3.0600	H15C···O3^x^	2.8500
C55···H4A^vii^	2.9200	H15C···H2B^vii^	2.3200
C56···H6A	2.7800	H15C···H2A^vi^	2.5800
C56···H6B	3.0900	H52···C55^i^	3.0600
C56···H4A^vii^	2.9500	H52···H5	2.3700
H2A···H11C	2.5600	H52···N12^iii^	2.7100
H2A···H15C^v^	2.5800	H53···H15A	2.3700
H2B···H6A	2.5900	H53···C53^viii^	2.9700
H2B···H11A	2.5000	H53···H53^viii^	2.4800
H2B···C15^vii^	3.0500	H55···C52^ix^	3.0600
H2B···H15C^vii^	2.3200	H55···O3^x^	2.7700
H4A···C51^vii^	3.0100	H56···C6	2.7200
H4A···C52^vii^	2.9800	H56···H4B^ix^	2.5700
H4A···C53^vii^	2.9300	H56···H6A	2.2100
H4A···C54^vii^	2.9400		
			
C2—C1—C6	109.04 (13)	C3—C4—H4B	109.00
C2—C1—C11	110.55 (14)	C5—C4—H4A	109.00
C2—C1—C12	108.69 (11)	C5—C4—H4B	109.00
C6—C1—C11	111.35 (12)	H4A—C4—H4B	108.00
C6—C1—C12	108.53 (13)	C4—C5—H5	108.00
C11—C1—C12	108.62 (14)	C6—C5—H5	108.00
C1—C2—C3	112.38 (13)	C51—C5—H5	108.00
O3—C3—C2	121.60 (17)	C1—C6—H6A	109.00
O3—C3—C4	122.51 (15)	C1—C6—H6B	109.00
C2—C3—C4	115.89 (12)	C5—C6—H6A	109.00
C3—C4—C5	112.38 (13)	C5—C6—H6B	109.00
C4—C5—C6	110.02 (12)	H6A—C6—H6B	108.00
C4—C5—C51	110.09 (12)	C1—C11—H11A	109.00
C6—C5—C51	113.81 (11)	C1—C11—H11B	109.00
C1—C6—C5	112.03 (11)	C1—C11—H11C	109.00
N12—C12—C1	178.70 (18)	H11A—C11—H11B	109.00
C5—C51—C52	119.32 (12)	H11A—C11—H11C	109.00
C5—C51—C56	122.83 (13)	H11B—C11—H11C	109.00
C52—C51—C56	117.83 (15)	C54—C15—H15A	109.00
C51—C52—C53	121.07 (12)	C54—C15—H15B	109.00
C52—C53—C54	121.23 (14)	C54—C15—H15C	109.00
C15—C54—C53	121.54 (16)	H15A—C15—H15B	109.00
C15—C54—C55	120.88 (16)	H15A—C15—H15C	109.00
C53—C54—C55	117.58 (17)	H15B—C15—H15C	109.00
C54—C55—C56	121.31 (13)	C51—C52—H52	119.00
C51—C56—C55	120.95 (14)	C53—C52—H52	119.00
C1—C2—H2A	109.00	C52—C53—H53	119.00
C1—C2—H2B	109.00	C54—C53—H53	119.00
C3—C2—H2A	109.00	C54—C55—H55	119.00
C3—C2—H2B	109.00	C56—C55—H55	119.00
H2A—C2—H2B	108.00	C51—C56—H56	120.00
C3—C4—H4A	109.00	C55—C56—H56	120.00
			
C6—C1—C2—C3	52.74 (16)	C4—C5—C51—C52	−88.85 (14)
C11—C1—C2—C3	175.47 (14)	C4—C5—C51—C56	89.28 (15)
C12—C1—C2—C3	−65.40 (19)	C6—C5—C51—C52	147.10 (12)
C2—C1—C6—C5	−58.85 (15)	C6—C5—C51—C56	−34.77 (17)
C11—C1—C6—C5	178.91 (12)	C5—C51—C52—C53	178.29 (12)
C12—C1—C6—C5	59.39 (14)	C56—C51—C52—C53	0.1 (2)
C1—C2—C3—O3	130.73 (15)	C5—C51—C56—C55	−179.06 (13)
C1—C2—C3—C4	−49.13 (17)	C52—C51—C56—C55	−0.9 (2)
O3—C3—C4—C5	−131.91 (13)	C51—C52—C53—C54	1.5 (2)
C2—C3—C4—C5	47.96 (15)	C52—C53—C54—C15	176.87 (13)
C3—C4—C5—C6	−51.08 (14)	C52—C53—C54—C55	−2.2 (2)
C3—C4—C5—C51	−177.30 (10)	C15—C54—C55—C56	−177.71 (14)
C4—C5—C6—C1	58.21 (15)	C53—C54—C55—C56	1.3 (2)
C51—C5—C6—C1	−177.70 (11)	C54—C55—C56—C51	0.2 (2)

Symmetry codes: (i) *x*, *y*−1, *z*; (ii) −*x*, *y*−1, −*z*+1/2; (iii) −*x*, −*y*+1, −*z*+1; (iv) −*x*, −*y*+2, −*z*+1; (v) *x*−1/2, *y*−1/2, *z*; (vi) *x*+1/2, *y*+1/2, *z*; (vii) −*x*, *y*, −*z*+1/2; (viii) −*x*+1/2, −*y*+3/2, −*z*+1; (ix) *x*, *y*+1, *z*; (x) −*x*, *y*+1, −*z*+1/2.

### Hydrogen-bond geometry (Å, °)

**Table:**

*D*—H···*A*	*D*—H	H···*A*	*D*···*A*	*D*—H···*A*
C4—H4A···Cg^vii^	0.99	2.61	3.5425 (15)	157

Symmetry codes: (vii) −*x*, *y*, −*z*+1/2.
